# Hardships in Italian Prisons During the COVID-19 Emergency: The Experience of Healthcare Personnel

**DOI:** 10.3389/fpsyg.2021.619687

**Published:** 2021-02-04

**Authors:** Ines Testoni, Giada Francioli, Gianmarco Biancalani, Sandro Libianchi, Hod Orkibi

**Affiliations:** ^1^Department of Philosophy, Sociology, Education and Applied Psychology (FISPPA), University of Padova, Padova, Italy; ^2^Emili Sagol Creative Arts Therapies Research Center, University of Haifa, Haifa, Israel; ^3^Medical Director of the Operative Unit of the Male and Female Imprisonment House in Rebibbia, and as President of the National Coordination of Operators for Health in Italian Prisons (Co.N.O.S.C.I.), Rome, Italy

**Keywords:** COVID-19, prison, burnout, working well-being, healthcare personnel, prison riots

## Abstract

**Background:** The recent COVID-19 pandemic has highlighted the deficiencies that characterize the functioning of the Italian national health system. Prisons have always mirrored the most radical expressions of these weaknesses. During the early stages of the pandemic, prison facilities across Italy underwent a series of changes dictated by the need to ensure the safety of the prisoners and staff. The adoption of these rules contributed to a total or partial redefinition of many central facets of life in prison, such as intake procedures for new arrivals and the ways prisoners were allowed to communicate with their families.

**Objectives:** The aim of this qualitative study was to analyze the testimony of penitentiary healthcare workers in prisons throughout Italy to determine the impact of COVID-19 on their professional and personal lives.

**Participants:** Thirty-eight participants were contacted and 20 decided to participate in the interview. The sample was made up of 10 women and 10 men. All the participants were members of the healthcare staff of a penitentiary facility (psychologists, psychiatrists, physicians, and nurses). All were recruited through an Italian association whose mission is the development, promotion, and implementation of social solidarity projects including prisoners' social and health care. This study was facilitated through representatives serving in nine different regions of Italy. The participants were divided according to their professional roles in prisons.

**Method:** In-depth interviews were conducted by telephone or online using telecommunication platforms (e.g., Zoom, WhatsApp, and Skype). The transcribed texts underwent thematic analysis using the Atlas.ti software to identify patterns of meaning across the dataset.

**Results:** Four main themes emerged from the analysis: Interpersonal difficulties, management and operational difficulties, the personal distress and bereavement of healthcare workers, and the distress of inmates. The importance of relationship management skills when interacting with prisoners emerged as a key topic in many interviews, and the participants highlighted the need for adequate training. The increase in prisoners' anxiety made communication more difficult.

**Conclusions:** The findings suggest that healthcare workers in jails need emergency-oriented training. Participants described their feeling of loneliness and quasi-abandonment when carrying out their duties during the pandemic. In particular, they underscored the need for psychological guidance to better manage altered reactions with prisoners and colleagues as a result of heightened death anxiety and isolation.

## Introduction

During the first months of 2020, the Italian health system was forced to grapple with the recent pandemic caused by the spread of COVID-19, which further undermined its endemic problems. The national health emergency significantly impacted life and work in prisons, including those of healthcare workers such as physicians, nurses, psychologists, and psychiatrists. In Italy, at the start of the pandemic, despite regional differences, the same solution was imposed throughout the country, including a systematic lockdown (Ministry of Health of Italy, 2020). These restrictive solutions negatively impacted specific sectors, including prisons, which are characterized by persistent and problematic overcrowding (Ristretti Orizzonti, 2020). To cope with the emergency, prevent infections in prison, and guarantee the safety of prisoners and personnel, the rules in force in prison communities were suddenly modified, including a ban on visits from relatives. Inmates could only contact their loved ones online by computer or tablet. These measures to reduce the risk of infection prompted a series of riots that broke out in many prisons on March 7–9, 2020. According to ANSA it (2020; an Italian news agency), in a single weekend, out of a total of 189 prisons, serious structural damage resulting from vandalism and arson affected over 70 prisons; in addition, 30 prisons held peaceful demonstrations (ANSA it, 2020). These riots in many cases enabled the inmates to gain access to restricted areas where drugs including certain lethal medications are stored. A total of 12 prisoners died during the uprisings. Roughly 70 prisoners escaped after internal attacks on structures and fires (Il Fatto Quotidiano, 2020). These dire events can be attributed to the abysmal conditions of Italian prisoners that stem from overcrowding, which makes for high constant stress levels of inmates and custodial workers. On the other hand, the prisoners' fear of being infected was linked to their frustration at being prevented from face to face encounters with their relatives.

These internal and external changes forced the healthcare professionals to rapidly modify their modes of intervention in the jails. In some cases, the personnel had to deal with the reactions of the inmates who had played a role (both major and minor) in these critical events.

We define a *critical event* as any situation that can severely challenge professionals who have to face a situation that requires skills they do not have, directly or indirectly, resulting in concern (Zamperini et al., 2015). A critical event is generally an unexpected event, given its low frequency (Gremler, 2004), which deprives professionals of the feeling of being in control of the situation and is characterized by a perception of danger for their psychological or physical well-being (Rotter, 1966). The term *critical service event* referred to any situation that could alter the rescuer's coping skills (Mitchell and Everly, 2001). This type of event constitutes a threat to the individual's well-being (Gremler, 2004; Testoni et al., 2019b). These factors are also classified by the Department of Penitentiary Administration at the Department of Justice and include calamities that can compromise the well-being of the prison community, such as the COVID-19 emergency (Ministry of Justice of Italy, 2011). This premise makes it possible to qualify the impact of a critical event based on the subjective perception of the people involved. Although the literature has dealt extensively with the level of stress of health professionals (Benedek et al., 2007) and critical incidents (e.g., Schluter et al., 2008; Brazil et al., 2010; Interculturel, 2017), there is no research on this specific issue. Half of the population considered the impact of the COVID-19 epidemic to be psychologically moderate or severe (Wang et al., 2020). However, the World Health Organization has identified healthcare workers as a job category that is at particular risk of developing a wide range of physical or psychological problems in the current pandemic situation (Koh et al., 2005). Stress, high workload, worries of contracting the infection or infecting one's family, the lack of adequate support in the workplace and the absence of effective supportive treatments, can negatively affect the well-being of healthcare workers (Moazzami et al., 2020; Vieta et al., 2020). Regarding the studies on the psychological effects of epidemics, a research by Salazar de Pablo et al. (2020) reports the most frequent symptoms in healthcare professionals, leaving, however, the prison context unexplored in this area. To respond to this need, the present study investigated the experiences and possible critical events in Italian prisons and their impact on healthcare personnel working in penitentiaries across Italy during the COVD-19 lockdown and emergency period.

## Method

### Aims

The purpose of this qualitative study (Seale et al., 2006) was to investigate the types of stressors, difficulties and the possible existence of critical events among health professionals in Italian prisons after the declaration of a state of emergency caused by the COVID-19 pandemic. Specifically, the goal was to determine the changes as perceived by the participants with respect to their own and the prisoners' well-being, the organizational climate, and the work done by these professionals to better understand the nature of the discomfort caused by the pandemic emergency.

### Participants and Procedure

The sample was composed of 20 participants (50% female) working in prison facilities throughout Italy: 6 in the North, 5 in the Center, and 9 in the South (75 physicians, 15 nurses, 10 psychologists, and 25% healthcare directors). The average age of the participants was 49 (range = 31–67 years; SD = 12). The average work years in the prison health sector was 19 years and all names reported are pseudonyms (see Table 1).

**Table 1:** Participants.

**Pseudonyms**	**Age**	**Profession**	**Years of working in the prison context**	**Years of working in their prison**
Rossella	41	Physician	16	9
Claudio	31	Physician	2	2 months
Donatella	54	Nurse	20	15
Andrea	34	Physician	5	1
Leonardo	31	Physician	2	2
Tiziana	36	Physician	6	6
Saverio	67	Healthcare Director	30	30
Livia	54	Psychologist	23	22
Silvia	48	Physician	23	3
Vittorio	61	Healthcare Director	35	30
Simona	35	Physician	7	4
Raffaella	57	Physician	4	3 months
Serena	55	Healthcare Director	35	10
Luca	56	Physician	30	10
Elio	49	Physician	20	11
Cristina	54	Nurse	21	15
Nicola	64	Healthcare Director	30	25
Sofia	60	Healthcare Director	33	4
Carlo	37	Physician	8	8
Ettore	55	Physician	28	6

The participants were contacted through a branch of the O.N.L.U.S.^1^ which coordinates healthcare professionals who work in jails all over Italy. The participants were informed of the research objectives and gave their informed consent before the interview. After agreeing to a date, the data were collected by telephone or *via* Zoom, Skype, or WhatsApp. This study was approved by the Padova University Ethics Committee for Experimentation (#BB4DCE00A75F9-FC621E922D1B98E00AB).

### Instruments and Data Analysis

The semi-structured in-depth interviews lasted about an hour. The aim was to explore prison healthcare workers' personal experiences, feeling, difficulties, strategies, and in particular the critical events they faced during the COVID-19 emergency. Participants described their lived experiences in the relational sphere, their perceptions of changes in the prisoners, and the ways the prison facility handled the emergency. A dialogue was developed by asking the participants to reflect on the effect of changes caused by the pandemic, the strategies implemented to face them, and the problems they could not solve.

After all the interviews had been recorded and transcribed, the texts underwent a thematic analysis (Braun and Clarke, 2006) to identify patterns of meaning regarding (Attride-Stirling, 2001) the difficulties faced by prison healthcare workers during the pandemic. The texts were processed using Atlas.ti, which made it possible to identify the logical connections in the texts. Atlas.ti optimizes the construction of a theoretical model based on text. The analysis of the text followed the six main phases outlined by Braun and Clarke (2006): preparatory organization; generation of categories or themes; coding data; testing emerging understanding; searching for alternative explanations; and writing up the report. Atlas.ti allows the development of a theoretical model firmly based on the text, to produce scientific knowledge by relating the researcher's categories of analysis with the meanings constructed by the subjects in the interview (Muhr, 1997). The analysis is conducted by attributing codes to significant portions of the text and the results are graphs of semantic networks, which describe the logical relationships between the narratives and categories identified by the researchers. As the analysis proceeded, the primary difficulties expressed by the participants emerged. The primary goal was to identify the changes prompted by the health emergency.

## Results

As shown in Table 2, the thematic analysis yielded four main themes: Interpersonal difficulties, management and operational difficulties, the personal distress and bereavement of healthcare workers, and the distress of inmates. Each theme was characterized by specific codes.

**Table 2:** Themes and main codes.

**Themes**	**Participants no. by region**	**Main codes**
Interpersonal difficulties	North: 6	Interpersonal difficulties between the healthcare and security personnel
	Center: 6	Interpersonal difficulties among healthcare personnel
	South: 7	
Management and operation difficulties	North: 6	Vague regulations
	Center: 5	Lack of training
	South: 4	Lack of resources
		Workload
Personal distress and bereavement of healthcare workers	North: 6	Anxiety attributed to Covid-19
	Center: 5	Difficulties related to the riots
	South: 7	Critical events
Distress of inmates	North: 6	Ban on face to face interactions with family
	Center: 5	Misperception of the outside world
	South: 7	Anxiety attributed to Covid-19
		Salience of death

### Interpersonal Difficulties

During the interviews, attention was paid to conflicts, clashes or interpersonal difficulties within the prison community during the pandemic, which was a significant source of distress for the interviewees. The interpersonal difficulties were delineated according to different dynamics that pointed to two main conflicts: interpersonal difficulties within the healthcare professionals, and clashes between the health professionals and the security (surveillance) personnel. With regard to the former, the onset of the pandemic generated conflicts within the health setting with colleagues. For example, Rossella, who is a physician, said “The climate has worsened a lot. I went back recently, but the climate is still very bad. Everything has gotten worse in the last two months. In short, a big mess!” (2:36). This perception was confirmed by Claudio, a continuity-of-care physician who reported “Perhaps the biggest difficulty I had was with my colleagues, I found it hard to collaborate with some, in the sense that a climate of fear had spread and some colleagues were highly affected. Discussions are unavoidable and it is not always possible to arrive at a consensus” (15:2) and Donatella, a nurse, “Some have been quite cowardly, because we have made choices in life anyway and so, yes, we are afraid, but it is the job that we have chosen, no one forced us. It is not fair to complain when things get difficult with the inmates, as some colleagues do” (18:3).

In addition, overall, there were repeated references to incidents between health professionals and the security personnel. There was excessive authoritarianism on the part of the latter toward the former, who at certain times felt they were treated the same as the prisoners. Andrea, a physician, said during the interview “Yes, the arrogance of the guards who refuse to wear masks, problems in taking people's temperature at the entrance. The security personnel or their representatives avoided the temperature check because they thought it was useless, they did not wear a mask, they did not take their temperature or asked not to as a favor. They had a different interpretation of the situation, they took it very lightly. Every time I asked them to respect the rules of hygiene, inevitably the discussion became conflictual” (17:11) and “The aggressiveness of the security staff toward us, young healthcare workers, is constant, but they have increased the pressure to get us fired. They have leveraged the fact that physicians are potentially greedy. Obviously, being aware of the ways in which the virus is transmitted, we have always shown that we are able to handle the situation safely for ourselves and for others, and to discredit this type of recrimination. However, each time we experience these situations as violent attacks on our professionalism” (17:9). Leonardo, an on-call physician, confirmed “It is very difficult to stand up to the arrogance of security personnel. It's like fighting a shark in the ocean, you can't do it” (4:9). Rossella said “Despite the COVID emergency and the importance of our work in this situation, we know that we can never afford the luxury of getting into a controversy with them because it would be a fight we would lose all down the line” (2:26).

### Management and Operational Difficulties

The failures in prison management due to the pandemic and the anxiety generated by the riots were hard for the participants. The initial lack of specific health and procedural guidelines for COVID-19 were mentioned by almost all the participants as a source of huge personal stress, given the impossibility of determining the responsibilities of the healthcare professionals and the penitentiary personnel (administrative officers and guards/wardens). The constant problems with management affected the participants' perceived self-efficacy and confidence in their job, as stated by Tiziana, a physician: “The greatest difficulty is… organizational mismanagement. We still don't have precise protocols defining the rules and the duties. Many issues between the health administration and the prison have not been resolved, so today we are in chaos. It is absolutely impossible to work serenely” (3:13). Saverio, a healthcare director, agreed, saying, “I felt very embarrassed, because there were times when I didn't know what to do, so I had to improvise, still thinking that it would very likely be wrong. The difficulties among colleagues and the other professionals made it impossible to find a solution through cooperation” (12:5). In some cases, this was associated with a perception of institutional inadequacy: Saverio said “Prisons depend on either the Ministry of Health or the Ministry of Justice. In Italy, neither ministry has defined specific steps to alleviate work-related stress. Maybe it is not easy, but it is important to show that the problem is being acknowledged” (12:13). Increased worries stemmed from the lack of health facilities for both professionals' and prisoners' detention, included quarantine areas. Livia, a psychologist, said: “What I felt as a health professional is that I did not feel protected. I, the physicians and nurses should have been the first to be checked and protected. This did not happen and we had to make do with it somehow” (5:24). Silvia, a physician, also stated “Throughout the month of February I worked with great anxiety and with great personal difficulties. I could not be effective, like many of my colleagues. We suffered for many reasons, and first of all because of the uncertainty caused by the lack of adequate health care facilities” (10:1). Vittorio, a healthcare director, stated “Prisons are increasingly overcrowded. The police made a whole series of arrests that they had left pending during the lockdown, so now there has been a sudden wave of new arrivals in the prisons, but the emergency is not over and we cannot think of handling them as we would have done before the pandemic” (7:20).

Many participants emphasized the lack of professional training, Rossella reported “We have had no training that prepares us to manage this kind of difficulty. Zero, zero, zero. Zero! We are always arguing about the usual bullshit, even about managing the actual risk of hepatitis. We are always talking about it, but in my opinion, we need to act in a more concrete way to understand the patients' diversity and how to manage it. There is no specific training here, either on a psychological level or at the level of emergency management!” (2:43); Vittorio said “I fought with the health company to ask for a certain amount of hours of specific professional training for people before putting them on their shift, but I am always rejected in the most absolute way. It is clear that there is an absolutely “do-it-yourself” introduction, without anything formal” (7:11); Sofia, a healthcare director, said “We do not have this training, it is something that was not taken into consideration. Penitentiary medicine has been in the national health system for a decade, so the health system has no trained people, there is no university exam, no post graduate training, no specialization. Now, in my opinion, there should be, to be able to do the work that I do and to be able to work with a patient in prison, because the patient who is also an inmate is not the same as a simple patient who is not in prison. As concerns the doctors, none of those who enter the prison actually know the patient-inmate, they know him in the field, which is completely another thing” (8:16).

### Personal Distress and Bereavement of Healthcare Workers

From a personal point of view, the participants' greatest concern was the fear of contracting COVID-19, which can be dangerous for themselves, their family members, and those who work or live inside the prison: Donatella “We were certainly afraid of getting sick, but also of a possible riot. I don't know what scared me more: the rebellion of the COVID infection. I didn't know how to manage this anguish” (18:4); Andrea “My fear was that the COVID outbreak would take everyone, including physicians, nurses, this was the thing that worried me the most” (17:1). Simona, a 35-year-old physician who has been working in prison for 7 years, said: “It has been difficult to manage the fear of infection, both as a physician and as a psychiatrist. I tried to make my fellow health professionals but also the inmates understand that this fear was normal and that knowing this could help us to manage it better. But it was really difficult and I don't know how effective it was. In addition, I also had to face my personal fear of the pandemic and its scale and the risk of infecting my family and loved ones” (9:13).

Participants who had been somehow involved in the riots reported that this experience severely impacted the climate of the entire facility. The testimony of Sofia also highlighted this change, “We have been experiencing precariousness in security, precariousness in conditions. Our offices were torched… we worked like we were in a war camp. We are still trying to adapt everything to these new conditions” (8:20); Sofia “Bad. It's something I prefer not to talk about, the worst thing that comes to my mind is the dead. The rest has been brought back, it has all returned slowly, the lights have been turned on again, the walls are being repainted, the carts have been bought back, a new armored car has been bought, but the dead are dead. They cannot return” (8:13).

The theme of death reappeared in the interviews, not only as it related to the pandemic, but also to the riots, as Sofia said: “No adequate measures were taken. If the authorities had come to see, they would have become aware of the seriousness of the situation. Instead they stayed outside, so they could not understand. Outside they only saw some of the events and certainly not as dramatically as we saw them, looking at the dead prisoners. From outside it was certainly not possible to perceive the levels of suffering that we had to endure inside. All the things that were destroyed in the uprising have been replaced, but they are things. And no one talks about those who died and what happened” (8:14). This argument also emerged in the testimony of Raffaella, a physician: “In my opinion, the revolt began because there were instigators who fomented the inmates. Thus, the prohibition of visits because of COVID was the spark. They broke through the grates, set fire to everything. They tore down the infirmary medicine cabinet, destroyed the radiology department. In a short time, they took over the whole prison. Luckily, they saved their medical records. Many died, some of them overdosed because they found psychotropic substances in the infirmary. Many were saved by physicians and nurses, who tried everything, had to improvise methods and instruments. But for many there was nothing anyone could do. And no one worked out any of this” (13:12). According to Simona, a physician: “All this happened because in prison there is no space to process fear, anxiety, loneliness, isolation. Here reigns the fear of dying of COVID separated from the rest of the world, the fear of dying alone. The most intense feeling is to be locked up and to die in a place isolated from the world” (9:10).

A perception of generalized mourning related to the prisoners who died during the riots appeared in all the narratives of those who worked in prisons where these took place. The generalized perception was that all the healthcare professionals had to manage their grief alone; however, Livia emphasized: “Generally, when I experience bereavement, I share my sadness with those with whom I have intimate relationships. In this pandemic, there was macro-social bereavement. I think it could have made people more capable of sharing their fears, worries, tragedies. This unstoppable series of tragedies increases solidarity or even just empathetic communication” (5:36).

### Distress of Inmates

The difficulties with the security personnel were considered one of the main causes of suffering by some inmates, because they are subjected, in opinion of some healthcare professionals, to possible dehumanization, as Leonardo, a physician, affirmed, “In my opinion a prison, for someone who has a medical background, is misleading after a while. The relationship that the personnel has with the prisoners can turn into a man-animal relationship and at times we too are forced to change our attitude” (4:11). According to Raffaella, a physician, however, not all prison guards behave the same way. In her point of view, “There are some very smart young people who try to establish a relationship that respects the dignity of the prisoner. By contrast, the older ones still think that prisoners are despicable beings, different from normal people and therefore it is inevitable that they have to suffer. It is part of their condition” (13:7).

However, the greatest difficulty during the pandemic emergency was the ban on direct communication with family members. This was considered the main factor underlying the inmates' manifestations of unease, even more than the fear of the virus. The initial deprivation of contact with their families was critical for the well-being of the prisoners who often openly manifested their unhappiness at times through violent actions: Livia, a psychologist, said “Because they are inmates, the first feeling they probably had of instability was the ban on their only contact with the outside world, the families, so much so that they did not realize, and we also had to contain them, which is why in our ward they did not riot… [we explained that] having a positive, asymptomatic person from their family come to the prison could create a problem for the whole prison” (5: 7); Donatella said “They got very angry about these additional restrictions of not being able to see family members. So, then we had to work a lot with the reassurances for their many questions like ‘what's going on?’, ‘How many deaths are there?’ And trying to explain the situation. We had to work a lot on this issue. Some understood the situation, others absolutely did not and were angry” (18:1); Ettore, a physician, said “At first, I noticed an increase in anger and in some cases of self-harm, but with the arrival of news from the outside they realized that it was not an arbitrary thing only toward them, but that it was a very serious situation” (14:5). These reactions were associated with a different perception of the world outside the prison that often did not allow them to understand the gravity of the pandemic in Italy, Leonardo said: “They realized that this was the case and they could not get out, seeing that even people could not leave the house they understood that it was normal that they also could not receive visitors. At first they were a bit more turbulent but then they understood” (4:18). Among the inmates, death was also significant, especially in terms of end-of-life within a prison without their family and loved ones: “It is precisely the fear of death that is the most important issue. It is useless to avoid speaking about it. All of us avoid this theme, and then we avoid talking about it with the inmates, because here everything is very difficult, unmanageable” (9:14).

Once again, the issue of managing the needs of inmates was considered relevant but also problematic. The fear of infection dominated, especially in relation to inmates with immunodeficiencies, as noted by Luigi, “Yes, the anxiety was very intense especially among HIV inmates, who live constantly in fear of infection. We couldn't reduce their anxiety because we did not know exactly how to do so” (5:11). Andrea said something similar: “Some inmates were really anxious about getting COVID especially those who had previous pathological conditions. All the prisoners were afraid, but some of them felt particularly exposed to the risk of dying” (17:2). Beyond the physical fear the prisoners were also obsessed with not being able to be with their loved ones in their moment of danger to defend them, as described by Livia: “The most worried were the new inmates, who are attached to their families and have not been in prison long, since February [2020] or shortly before. They feel very guilty toward their relatives, because they fear that they have left their loved ones alone in their time of need. They fear that something will happen to them and that they will not be able to help them” (5:12).

## Discussion

This study examined the difficulties of health professionals in Italian prisons in different regions during the first phase of the COVID-19 outbreak. Although the situation of Italian prisons varies significantly, there were obvious similarities. Thus the pandemic revealed some generalized problems with respect to the prison issue, because there were no significant differences in the impact of the emergency across the three areas in the North, Center, and Southern Italy. The most significant difficulty presented by all participants was overcrowding, a phenomenon that existed before the pandemic (Ministry of Justice of Italy, 2011; Ristretti Orizzonti, 2020; Testoni et al., 2020a,b), but this was not the only one. Lack of competences, organization, and facilities were consistently denounced by almost all participants who considered that the hardships they faced are rooted in these institutional weaknesses. The sudden perception of lack of confidence on the job due to exposure to the risk, confusion related to rules and relational status, the absence of specific protocols undermined the perceived self-efficacy of participants, consistent with the literature on critical events (Testoni et al., 2018, 2019a).

One specific issue was the conflict between the healthcare professionals and the security personnel. The lack of clear behavioral guidelines led to hierarchical conflicts in terms of adhering to social distancing and mask rules. The lack of compliance on the part of the security personnel was paralleled by the inability of the healthcare professionals to enforce these rules. The participants considered that contradictory regulations added to their level of distress. These issues highlight the need for an organizational reformulation that defines more accurately the duties and the areas of competence of health workers and prison guards. It is hoped that the standardization of these aspects will contribute to improving the management methods, which are excessively general, which were reported as critical by the interviewees. Many stated that those who suffered the most from the situation were the inmates. The participants described the discomfort experienced by the inmates, including being cut off from their families, their distorted perception of the world outside, and their anxieties related to death. As noted in the literature, the relationship between prison guards and inmates is characterized by tensions that can result in dehumanization (Testoni et al., 2020b). Some participants, in interviews, referred to high levels of dehumanization in this relationship, comparing the relationship between guards and inmates with that between humans and animals.

Most respondents stated that one way of dealing with these uncertainties would be specific training courses. The perceived lack of competence permeated all four key themes. They stressed the need for adequate training and support to healthcare professionals, which would give them the means to cope with critical events during the pandemic and reduce the risk factors identified in this this study.

The analysis also revealed the need to deal coherently with the fear of death and mourning (Testoni, 2016). The lack of personal protective equipment was associated with the perception that the institution had abandoned its healthcare professionals. This was associated with the fact that the institution provided no support system for coping with anxiety, grief, and bereavement, which is crucial to processing the pandemic. In particular, the riots were interpreted as an expression of the prisoners' already high level of stress coupled with the frustration of not being able to speak and meet directly with relatives and friends. The lack of specific competencies in this regard made the participants feel helpless, but also left alone to manage the trauma of mourning for those who died during the uprisings. A potential limitation of this qualitative study is the non-representativity of the results since it only involved a small number of penitentiary institutions in Italy. A further potential limitation is that the difficulties experienced by the security personnel in the same institutions were not taken into account or compared to those reported by the healthcare professionals. Future research could therefore be extended to all penitentiary institutions using validated distress-work-related scales accompanied by a questionnaire with open questions, involving both healthcare and security personnel. Intervention programs could include experiential workshops to promote self-control, perspective taking, personal strengths, and hope (Azoulay and Orkibi, 2015; Orkibi, 2019; Orkibi and Feniger-Schaal, 2019; Feniger-Schaal and Orkibi, 2020). Further future studies, in order to deepen the results that emerged from the research also on a higher number of interviewees, will help to make it possible to identify specific psychological interventions and management changes necessary to improve the well-being of the entire prison community.

## Conclusion

This study was designed to pinpoint the issues that have had the greatest impact on the well-being of penitentiary healthcare workers during the COVID-19 emergency. The influence that each exerts on the others is undeniable, thus overall delineating the risk factors affecting prison healthcare staff. The lack of adequate-specific training in prison for health workers was crucial and was expressed in all four themes, as well as the management of detainee patients, whose specific needs, if not treated adequately, may exacerbate their already high distress, thus triggering episodes of revolt and violence. One of the main factors that may be related to prisoner aggression was the sudden deprivation of direct contact with family members, combined with their lack of perception of COVID-related events in the outside world.

The theme of death and the anxieties that affected all those in the prison impacted all the relationships. The healthcare professionals interviewed here perceived this, but felt ill- equipped to deal with this problem.

## Data Availability Statement

The raw data supporting the conclusions of this article will be made available by the authors, without undue reservation.

## Ethics Statement

The studies involving human participants were reviewed and approved by Ethical Committee for the Psychological Research of the University of Padova. The patients/participants provided their written informed consent to participate in this study.

## Author Contributions

IT: research design and project planning, supervision of the research, analysis of the texts, methodology, and article writing. GF: data collection, analysis of the texts, and article writing. GB: methodology, analysis of the texts, and article writing. SL: research design and project planning. HO: article writing. All authors contributed to the article and approved the submitted version.

## Conflict of Interest

The authors declare that the research was conducted in the absence of any commercial or financial relationships that could be construed as a potential conflict of interest.

## Footnotes

^1^ONLUS, organizzazione non lucrativa di utilità sociale: Non-profit Organization of Social Utility.

## References

[B1] ANSA it (2020, May 8). The Prison Revolt in March, WHO Was Behind the Riots—Legality and School (La rivolta Delle carceri a marzo, CHI c'era dietro I disordini—Legalità and Scuola). ANSA.it. Available online at: https://www.ansa.it/canale_legalita_scuola/notizie/2020/05/08/la-rivolta-delle-carceri-a-marzo-chi-cera-dietro-i-disordini_9bc30fe7-c189-4e82-b567-ad422a00719d.html (accessed March 08, 2020).

[B2] Attride-Stirling J. (2001). Thematic networks: an analytic tool for. Qual. Res. 1, 385–405. 10.1177/146879410100100307

[B3] Azoulay B. Orkibi H. (2015). The four-phase CBN psychodrama model: a manualized approach for practice and research. Arts Psychother. 42, 10–18. 10.1016/j.aip.2014.12.012

[B4] Benedek D. Fullerton C. Ursano R. (2007). First responders: mental health consequences of natural and human-made disasters for public health and public safety workers. Annu. Rev. Public Health 28, 55–68. 10.1146/annurev.publhealth.28.021406.144037 17367284

[B5] Braun V. Clarke V. (2006). Using thematic analysis in psychology. Qual. Res. Psychol. 3, 77–101. Tratto da. Available online at: http://www.informaworld.com/10.1191/1478088706qp063oa (accessed 2006).

[B6] Brazil K. Kassalainen S. Ploeg J. Marshall D. (2010). Moral distress experienced by health careprofessionals who provide home-based palliative care. Soc. Sci. Med. 71, 1667–1791. 10.1016/j.socscimed.2010.07.032 20832923

[B7] Feniger-Schaal R. Orkibi H. (2020). Integrative systematic review of drama therapy intervention research. Psychol. Aesthet. Creat. Arts 14, 68–80. 10.1037/aca000025730779787

[B8] Gremler D. D. (2004). The critical incident technique in service research. J. Serv. Res. 7, 65–89. 10.1177/1094670504266138

[B9] Il Fatto Quotidiano (2020, March 10). Coronavirus, Prisons In Revolt: 12 Victims. New Riots in Some Prisons. [Coronavirus, carceri in rivolta: 12 vittime. Nuovi disordini in alcuni penitenziari]. Available online at: https://www.ilfattoquotidiano.it/2020/03/10/coronavirus-carceri-in-rivolta-altri-3-detenuti-morti-a-rieti-nuove-proteste-a-siracusa-e-caserta-a-foggia-evasione-di-massa-23-ricercati-la-procura-di-milano-apre-inchiesta-sulla-sommossa-a-san/5730183/ (accessed March 10, 2020).

[B10] Interculturel E. (2017). Catalog of Critical Incidents [Catalogo degli Incidenti critici]. Healthy Diversity | Intercultural competences for the health sector Available online at: https://healthydiversity.eu/media/head-catalogo-degli-incidenti-critici.pdf

[B11] Koh D. Lim M. K. Chia S. E. Ko S. M. Qian F. Ng V.. (2005). Risk perception and impact of severe acute respiratory syndrome (SARS) on work and personal lives of healthcare workers in Singapore. Med. Care 43, 676–682. 10.1097/01.mlr.0000167181.36730.cc 15970782

[B12] Ministry of Health of Italy (2020). Covid-19—Situation in Italy [Covid-19—Situazione in Italia]. Available online at: http://www.salute.gov.it/portale/nuovocoronavirus/dettaglioContenutiNuovoCoronavirus.jsp?area=nuovoCoronavirusandid=5351andlingua=italianoandmenu=vuoto (accessed February, 2020).

[B13] Ministry of Justice of Italy (2011). Operational Management of Critical Situations [Gestione operativa delle situazioni critiche]. Department of Penitentiary Administration, Italy.

[B14] Mitchell J. T. Everly G. S. (2001). Critical Incident Stress Debriefing: An Operations Manual for CISD, Defusing, and Other Group Crisis Intervention Services. Ellicott City, MD: Chevron Publishing.

[B15] Moazzami B. Razavi-Khorasani N. Dooghaie Moghadam A. Farokhi E. Rezaei N. (2020). COVID-19 and telemedicine: Immediate action required for maintaining healthcare providers well-being. J. Clin. Virol. 126:104345. 10.1016/j.jcv.2020.104345 32278298 PMC7129277

[B16] Muhr T. (1997). Atlas.ti Short User's Guide. Berlin: Scientific Software Development.

[B17] Orkibi H. (2019). Positive psychodrama: a framework for practice and research. Arts Psychother. 66:101603 10.1016/j.aip.2019.101603

[B18] Orkibi H. Feniger-Schaal R. (2019). Integrative systematic review of psychodrama psychotherapy research: trends and methodological implications. PLoS ONE 14:e0212575. 10.1371/journal.pone.0212575 30779787 PMC6380607

[B19] Ristretti Orizzonti (2020, Marzo 27). Hurry up: Avoid the Covid Apocalypse in Our Prisons [Fate presto: evitate l'apocalisse da Covid nelle nostre carceri]. Ristretti Orizzonti: Available online at: http://www.ristretti.org/index.php?option=com_contentandview=articleandid=88595:qfate-presto-evitate-lapocalisse-da-covid-nelle-nostre-carceriqandc (accessed March 27, 2020).

[B20] Rotter J. (1966). Generalized expectancies for internal versus external control of reinforcement. Psychol. Monogr.Gen. Appl. 80, 1–28. 10.1037/h0092976 5340840

[B21] Salazar de Pablo G. Vaquerizo-Serrano J. Catalan A. Arango C. Moreno C. Ferre F.. (2020). Impact of coronavirus syndromes on physical and mental health of health care workers: systematic review and meta-analysis. J. Affect. Disord. 275, 48–57. 10.1016/j.jad.2020.06.022 32658823 PMC7314697

[B22] Schluter J. Seaton P. Chaboyer W. (2008). Critical incident technique: a user's guide for nurse researchers. J. Adv. Nurs. 61, 107–114. 10.1111/j.1365-2648.2007.04490.x 18173737

[B23] Seale C. Gobo G. Gubrium J. Silverman D. (eds.). (2006). Qualitative Research Practice. London: SAGE Publications.

[B24] Testoni I. (2016). *The Psychology of Death and Mourning: From Clinical Work to Death Education* [Psicologia del lutto e del morire: Dal lavoro clinico alla death education] Psychother. Hum. Sci. 24, 229–252. 10.3280/PU2016-002004

[B25] Testoni I. Carafa M. L. Bottacin M. Zamperini A. Galgani M. (2018). The nursing hospice care: critical incidents in managing the relationship with patients and their families [L'assistenza infermieristica in hospice: incidenti critici nella gestione della relazione con pazienti e familiari]. Prof. Inferm. 71, 151–159. 10.7429/pi.2018.713151 30457269

[B26] Testoni I. Marrella F. Biancalani G. Cottone P. Alemanno F. Mamo D.. (2020a). The value of dignity in prison: a qualitative study with life convicts. Behav. Sci. 10:95. 10.3390/bs10060095 32481496 PMC7349769

[B27] Testoni I. Nencioni I. Ronconi L. Alemanno F. Zamperini A. (2020b). Burnout, reasons for living and dehumanisation among Italian penitentiary police officers. Int. J. Environ. Res. Public Health 17:3117. 10.3390/ijerph17093117 32365763 PMC7246835

[B28] Testoni I. Pesci S. De Vincenzo C. Dal Corso L. Zamperini A. (2019a). Work and spirituality among people with asperger syndrome: an exploratory study. J. Disabil. Relig. 23, 178–196. 10.1080/23312521.2019.1580174

[B29] Testoni I. Pompele S. Venturini D. Zanca G. Maccarini A. M. (2019b). The role of support administration: a study on critical incidents. J. Health Hum. Serv. Adm. 42, 206–256.

[B30] Vieta E. Perez V. Arango C. (2020). Psychiatry in the aftermath of COVID-19. Rev. Psiquiatr. Salud. Ment. 13, 105–110. 10.1016/j.rpsmen.2020.04.004 38620300 PMC7177054

[B31] Wang C. Pan R. Wan X. Tan Y. Xu L. Ho C. S.. (2020). Immediate psychological responses and associated factors during the initial stage of the 2019 coronavirus disease (COVID-19) epidemic among the general population in China. Int. J. Environ. Res. Public Health 17:1729. 10.3390/ijerph17051729 32155789 PMC7084952

[B32] Zamperini A. Paoloni C. Testoni I. (2015). The emotional labor of nursing: critical incidents and coping strategies [Il lavoro emozionale dell'assistenza infermieristica: Incidenti critici e strategie di coping]. Assitenza Inferm. Ric. 34, 142–148. 10.1702/2038.22142 26488930

